# Trained Immunity-Based Vaccines: A New Paradigm for the Development of Broad-Spectrum Anti-infectious Formulations

**DOI:** 10.3389/fimmu.2018.02936

**Published:** 2018-12-17

**Authors:** Silvia Sánchez-Ramón, Laura Conejero, Mihai G. Netea, David Sancho, Óscar Palomares, José Luis Subiza

**Affiliations:** ^1^Department of Clinical Immunology and IdISSC, Hospital Clínico San Carlos, Madrid, Spain; ^2^Department of Immunology, ENT and Ophthalmology, Complutense University School of Medicine, Madrid, Spain; ^3^Inmunotek, Alcalá de Henares, Spain; ^4^Department of Internal Medicine and Radboud Center for Infectious Diseases, Radboud University Medical Center, Nijmegen, Netherlands; ^5^Department for Genomics and Immunoregulation, Life and Medical Sciences Institute, University of Bonn, Bonn, Germany; ^6^Immunobiology Laboratory, Centro Nacional de Investigaciones Cardiovasculares, Madrid, Spain; ^7^Department of Biochemistry and Molecular Biology, School of Chemistry, Complutense University of Madrid, Madrid, Spain

**Keywords:** adjuvants, innate immunity, immunostimulants, pattern recognition receptors (PRRs), PRR-ligands, trained immunity, trained immunity-based vaccines (TIbV), vaccines

## Abstract

Challenge with specific microbial stimuli induces long lasting epigenetic changes in innate immune cells that result in their enhanced response to a second challenge by the same or unrelated microbial insult, a process referred to as trained immunity. This opens a new avenue in vaccinology to develop *Trained Immunity-based Vaccines* (TIbV), defined as vaccine formulations that induce training in innate immune cells. Unlike conventional vaccines, which are aimed to elicit only specific responses to vaccine-related antigens, TIbV aim to stimulate broader responses. As trained immunity is generally triggered by pattern recognition receptors (PRRs), TIbV should be formulated with microbial structures containing suitable PRR-ligands. The TIbV concept we describe here may be used for the development of vaccines focused to promote host resistance against a wide spectrum of pathogens. Under the umbrella of trained immunity, a broad protection can be achieved by: (i) increasing the nonspecific effector response of innate immune cells (e.g., monocyte/macrophages) to pathogens, (ii) harnessing the activation state of dendritic cells to enhance adaptive T cell responses to both specific and nonrelated (bystander) antigens. This capacity of TIbV to promote responses beyond their nominal antigens may be particularly useful when conventional vaccines are not available or when multiple coinfections and/or recurrent infections arise in susceptible individuals. As the set of PRR-ligands chosen is essential not only for stimulating trained immunity but also to drive adaptive immunity, the precise design of TIbV will improve with the knowledge on the functional relationship among the different PRRs. While the TIbV concept is emerging, a number of the current anti-infectious vaccines, immunostimulants, and even vaccine adjuvants may already fall in the TIbV category. This may apply to increase immunogenicity of novel vaccine design approaches based on small molecules, like those achieved by reverse vaccinology.

## Background

Conventional anti-infectious vaccines are primarily intended to target specific pathogens by enhancing an antigen-specific adaptive immune response. This response is based on triggering B and T lymphocytes that, by virtue of their clonally segregated antigen receptors, generate effector, and memory cells. Proliferation and differentiation of specific lymphocytes is the basis of immunological memory, a hallmark of the adaptive immune response and the rationale behind conventional vaccines. Louis Pasteur built on the work of Edward Jenner to develop the principles of using attenuated live microbes to prevent the pathogen's caused disease (1). Pre-exposure vaccination was a major breakthrough in the prevention of many infectious diseases. His rabies vaccine for post-exposure prophylaxis in the severely ill boy Joseph Meister in 1885 raised the concept of therapeutic vaccines (2).

Immunological adaptive memory has been traditionally defined as long-term acquired memory against an encountered antigen through infection or immunization, leading to a quicker and heightened immune response upon an ulterior *rendezvous* (3). Resting clones of memory B and T cells can survive at different compartments for several decades until reactivation by recall responses (4, 5). Recent epidemiological studies highlight the role of subclinical infections or repeated endemic exposure for the maintenance of protective antigen-specific antibodies and T cells, indicating the dependency of this adaptive memory on antigen-re-exposure (6, 7). Besides, the persistent specific T and B lymphocyte activation can also favor “infectious immunity,” a process by which innate immune responses are enhanced by mechanisms depending on the persistence of the activation of adaptive immunity (3).

### Trained Immunity

Importantly however, solid epidemiological data have also demonstrated that certain mild infections or vaccinations, such as with bacilli Calmette-Guerin (BCG), lead to protection against heterologous infections, with a strong impact on overall mortality due to infection for up to 1 year (8–11). When vaccination against smallpox was introduced around 200 years ago, positive side-effects such as protection against measles, scarlet fever and whooping cough, among others, were noticed (12). These and many other clinical observations, pointed to a long-lasting non-specific collateral benefit associated to these vaccines, regardless of specific priming and subsequent clonal selection of T and B lymphocytes specific for the nominal antigens present in the vaccine. In recent years, it has become evident that cells of the innate immunity may be primed upon encounter with certain pathogens or molecular patterns associated to pathogens (PAMPs), acquiring a higher resistance to a second infection against the same or unrelated pathogens (cross-protection) for a relatively long time (13, 14). This concept has been termed “trained (innate) immunity.” Trained immunity implies adaptation of innate immunity processes in a *de-facto* innate immunological memory, and plays an essential role in vertebrates (15), which is similar to that described for bacteria, plants and invertebrates (16).

Mechanistically, trained immunity is defined by immunological, metabolic and epigenetic hallmarks (17–20). Several studies have shown that metabolic reprogramming through a shift from oxidative phosphorylation to aerobic glycolysis (the Warburg effect) mediated by the Akt/mTOR/HIF-1α pathway is a key mechanism for trained immunity responses (21, 22). The glycolysis, glutaminolysis, and cholesterol synthesis pathways in monocytes and macrophages were identified as the essential underlying mechanism linking epigenetic rewiring and the induction of improved innate immunity (22, 23). Thus, changes in cellular metabolism influence the epigenetic reprogramming of innate immune cells having an impact on cytokine and reactive oxygen species' production. In this regard, trained immunity regulates epigenetic changes such as H3K4 trimethylation and H3K27 acetylation, both associated with active chromatin, and H3K9 trimethylation, a repressive marker (19, 22, 24). Trained immunity favors the production and release of proinflammatory cytokines such as TNF-α, IL-6 and IL-1β by innate immune cells upon exposure to a second stimulus (14, 24, 25). Most of these features differ from what classically has been postulated for the innate immune system, as trained immunity induces functional reprogramming within innate immune cells that maintain these cells in a “ready-to-react” functional state over extended periods of time. Interestingly, although a maximum duration of trained immunity effects has been reported up to 3 months (24), a long lasting effect of trained cells with the capacity to enhance T cell responses up to 1 year is feasible (26), thus bridging innate training with adaptive responses. Moreover, the storage of specific long peptides for ulterior long-lasting cross-presentation to elicit cytotoxic T lymphocytes is an additional feature that may be associated to trained immunity in monocytes and could bridge innate and adaptive imprinting (27).

A striking example indicating a durable change within the innate immunity compartment is the imprinting of BCG on bone marrow hematopoietic stem cells and multipotent progenitors, giving rise to epigenetically modified macrophages that provide better protection against virulent *M. tuberculosis* than naïve macrophages (28). Unlike the classical memory following the adaptive immune response, long-term responses associated to trained immunity are not based on a clonal expansion of lymphocytes but on reprogramming myeloid cells by stable epigenetic changes (Table 1).

**Table 1:** Similarities and differences between trained and adaptive immunity.

**Feature**	**Trained immunity**	**Adaptive immunity**
Specificity	±	+++
Inducers	Pathogens and derived products (PAMPs)	Antigen presentation plus costimulatory signals and cytokines from DCs
Receptors	PRRs	sIg, TCR, receptors for costimulation and cytokines
Clonality	No	Yes
Cells	Monocytes, Macrophages, NK, DCs, ILCs, and other innate immune cells	B and T lymphocytes
Memory	Months	Years
Memory mechanism	Epigenetic modifications	Clonal expansion and differentiation

Pathogen recognition receptors (PRRs) expressed on innate immune cells, including long-lived macrophages and their precursors, are involved in the stimulation of trained immunity. Different PRRs have been involved in this task, such as C-type lectin receptors (CLRs) and Nod-like receptors (NLRs). Training of the innate immunity is therefore based on boosting non-specific immunity to re-infection by bacteria, fungi or viruses by certain pathogen's derived components (19). There are many examples of pathogen-associated molecules with evidence of cross-protection in experimental models (Table 2). The increased host defense induced by trained immunity, while effective against a range of pathogens, is non-specific as it is mediated by the release of proinflammatory cytokines such as IL-1α and TNF-α and/or reactive oxygen species (ROS) (17). The role of IL-1α in host resistance to infection and how TNF-α protects against infections have been recently updated (39, 40). Higher resistance due to trained immunity does not mean however an absolute resistance to every type of second infection that, on the other hand, may be favored by facts beyond the innate immune response. This might account for why a natural infection, such as primary infection by influenza A virus, can result in bacterial pneumonia resulting from superinfection by *Streptococcus pneumoniae* or other bacterial strains.

**Table 2:** Examples of pathogen-associated molecules with experimental evidence of cross-protection.

**Component**	**Source**	**Cross-protection**	**References**
LPS (endotoxin)	Most Gram-negative bacteria, such as *E. coli*	*Staphylococcus aureus*	(29)
Peptidoglycan component muramyl dipeptide	Bacteria	Toxoplasma	(30)
Flagellin	Gram-negative bacteria	Gram-positive bacterium *Streptococcus pneumoniae*	(31)
		Rotavirus	(32)
FimH	*E. coli*	Influenza virus	(33)
β-glucan	Fungi	*Staphylococcus aureus Streptococcus pneumoniae*.	(34)
Chitin	Fungi	*Staphylococcus aureus* or *Escherichia coli*	(35)
CpG oligodeoxynucleotide	Bacteria, synthetic	*E. coli*	(36)
		Influenza virus	(37)
			(38)

## Moving Ahead Conventional Vaccines: Trained Immunity-Based Vaccines (TIbV)

The exploitation of the principles of trained immunity may result in a next generation of anti-infectious vaccines (41–43). Trained immunity-based vaccines (TIbV) may confer a broad protection far beyond to the nominal antigens they contain. By proper targeting of innate immune cells to stimulate trained immunity, both nonspecific and specific immune responses can be enhanced by TIbV. Such responses can also be driven against bystander pathogens encountered by the host during the window of trained immunity.

Vaccines using attenuated and/or inactivated pathogens may be examples of TIbV as long as they contain PAMPs able to trigger PRRs inducing trained immunity. Different PRR ligands have been described as trained immunity stimuli, like *Candida*-derived β-glucan or BCG-derived muramyl dipeptide, triggering CLRs (dectin-1) or NLRs (NOD2), respectively. Of note, by using these training stimuli, slight differences on how they modify the cellular metabolism of innate immune cells has been described (22). This opens the possibility that different trained immunity outcomes may be achieved varying the set of PRR ligands used in the TIbV. At this point, it should be noted that, within the context of a vaccine, the fact that trained innate immune cells may enhance adaptive responses is essential. In this regard, the role played by DCs, the cellular link between innate and adaptive immunity (44), must be pivotal. Although trained immunity is linked to innate cells such as monocytes, macrophages and NK cells (19), the trained immunity-promoting BCG vaccine can also enhance heterologous T cell responses (26, 45, 46). It has been speculated that the increased expression of certain PRR in innate trained cells, as well as the release of typical innate immunity cytokines, such as IL-1β, contribute to enhance adaptive T cell responses (26). In this regard, the polybacterial sublingual vaccine MV130, which contains whole cell heat-inactivated bacteria, has been shown to enhance *in vivo* T cell responses to unrelated antigens, while priming DCs and inducing IL-1β release *in vitro* (47). DCs with high immunostimulatory properties that enhance adaptive immune responses via IL-1β release have been described (48). Moreover, the role of inflammasome-associated IL-1 family cytokines in delineating the adaptive immune responses is established regarding differentiation of Th17 cells and promoting effector functions of Th1 cells (49). Interestingly, these DCs are rendered “hyperactive” by releasing IL-1β in absence of cell death by virtue of an alternative inflammasome pathway modulated by certain TLR ligands of microbial origin, like LPS and peptidoglycans (49). Thus, strong adaptive immune responses may be stimulated by certain PRRs stimuli that may complement those inducing trained immunity.

One of the most interesting aspects of trained immunity is that innate immune cells maintain a primed functional state for a quite long period of time (50). As it may last more than several months (26), the enhanced immune responses induced by a given TIbV may also be applied to possible bystander pathogens encountered by the host during this time frame. Thus, another relevant aspect of TIbV is that they may promote both nonspecific and specific resistance to unrelated pathogens while trained immunity is still present. This may be of particular interest when co-infections with the pathogens included in the TIbV are likely, especially in the context of recurrent infections. Thus, under the umbrella of trained immunity, the TIbV concept emerges as a new paradigm of vaccines seeking to increase host resistance against a broad spectrum of pathogens.

**Figure 1:**
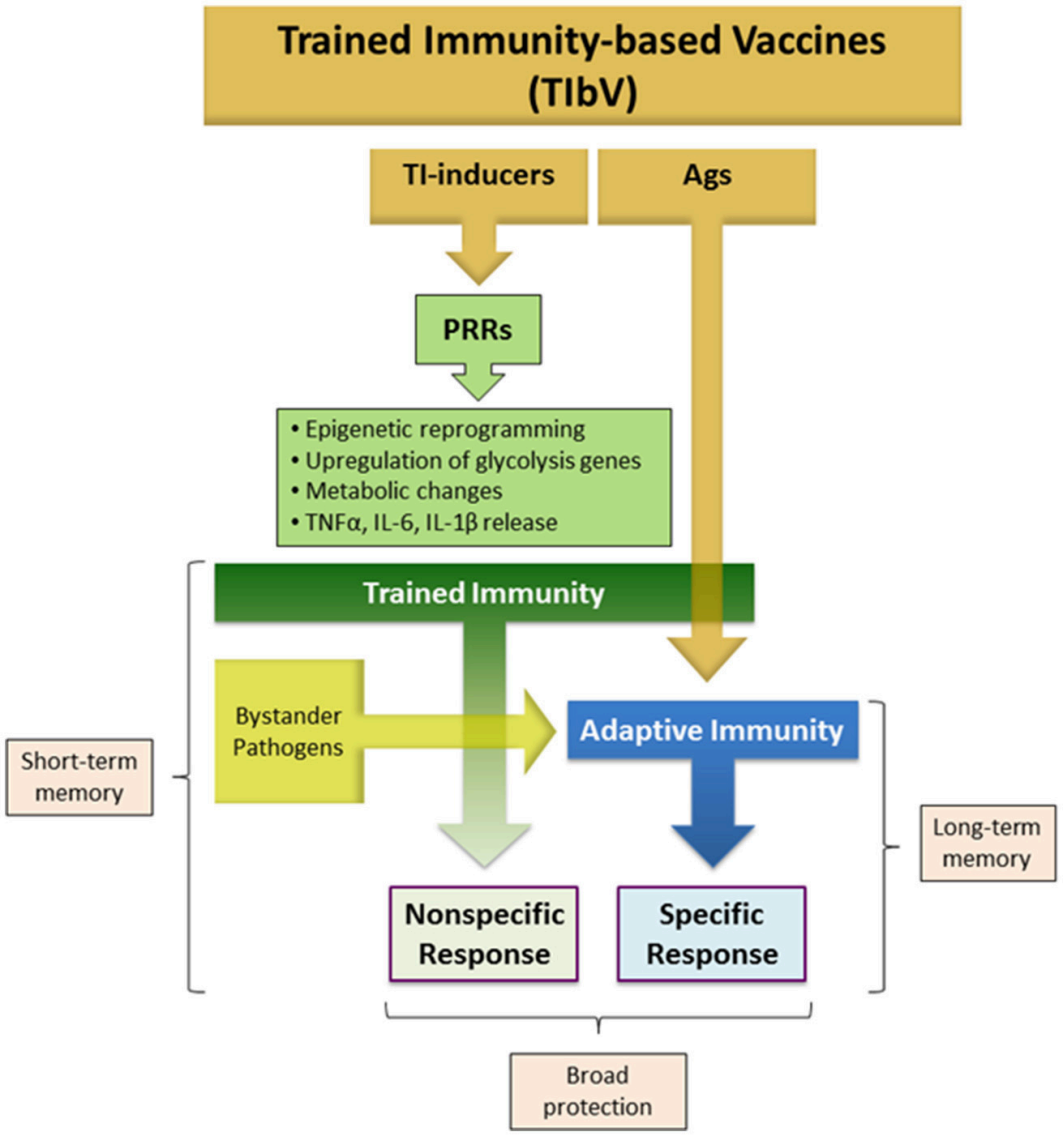
Trained immunity-based vaccine components. TIbV consist of two essential components: (a) Trained immunity (TI) inducers: a range of PAMPs that target a variety of PRRs triggering different signaling pathways that mediate trained immunity. (b) TIbV-related Ags: the antigens associated with the pathogens acting as TI-inducers to which an adaptive immunity is aimed. Thus, TIbV are characterized by conferring Ag-nonspecific resistance directly dependent on trained immunity stimulation plus an Ag-specific resistance dependent on adaptive immunity against the TIbV components and eventual bystander pathogens. PAMP, pathogen-associated molecular pattern; PRR, pattern recognition receptor; Ag, antigen.

With the above concepts in mind, the proposed term of TIbV can be applied for those anti-infectious vaccines composed of whole microorganisms or derived products that display the following features (Figure 1):

- Composed of trained immunity inducers, i.e. certain PAMPs, in addition to pathogen specific antigens.
- Intended to be effective not only against the specific pathogens targeted by the vaccine, but also against heterologous pathogens.

In contrast to conventional vaccines, TIbV efficacy cannot be measured solely in terms of specific responses to the nominal antigens included in the vaccine. In this regard, the clinical outcome scored by lower infectious rates in particular clinical settings is necessary.

### Examples of Non-conventional Vaccines That Can be Ascribed as TIbV

**Figure 2:**
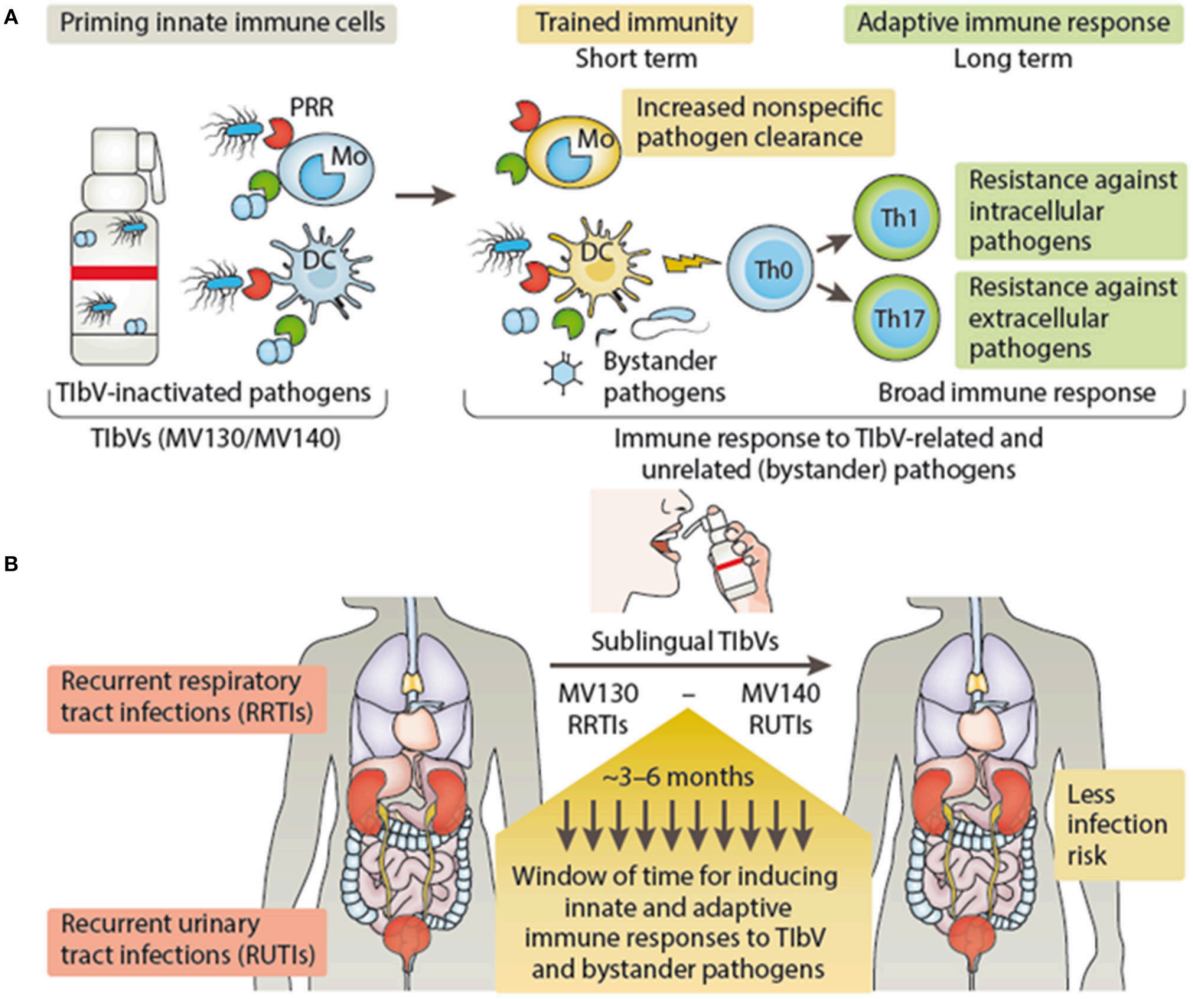
Trained immunity-based vaccine mechanisms of action **(A)** and clinical outcome **(B)**. (A) TIbV act on the cells of the innate immune system, such as macrophages/monocytes and DCs inducing trained immunity which in turn will lead to nonspecific resistance and pathogen clearance. In addition, trained DCs enhance T cell responses and T helper differentiation (e.g., Th1 and Th17) against TIbV-related and unrelated (bystander pathogens) antigens. (B) In the context of recurrent respiratory or urinary tract infections, TIbV have the potential to induce a protective period of time providing the host resistance against TIbV-related and bystander pathogens during this frame time, reducing the infection rate. DC, dendritic cells; Mo, monocyte; PRR, pattern recognition receptor; Th, T helper cell; Th0, naïve T cells; TIbV, trained immunity-based vaccine. MV130, polybacterial vaccine containing whole cell heat-inactivated bacteria that produce frequent infections in the respiratory tract. MV140, polybacterial vaccine containing whole cell heat-inactivated bacteria that produce frequent infections in the urinary tract.

Vaccines that may fall in the category of TIbV include those bacterial preparations used for recurrent infections for either the respiratory or urinary tract (51–53). Previous and recent studies provide many clinical observations that combinations of inactivated bacterial vaccines induce cross-protection against infections produced by quite different microorganisms (41, 54). In the case of the sublingual vaccine MV130, designed to prevent recurrent respiratory tract infections, a significant reduction in patient's rate of infection was observed (51). Besides inducing specific T cell immunity against the bacteria included in MV130, treated patients showed an enhancement in T cell response to unrelated flu antigens (51). MV130 triggers TLR and NLR signaling pathways on DCs releasing trained immunity hallmark cytokines (TNFα, IL-6, and IL-1β) (47). In addition, MV130 promoted the generation of Th1 and Th17 responses with high levels of IL-10 both *in vitro* and *in vivo* to MV130-related and bystander antigens (47). A recent clinical trial performed in children with recurrent wheezing attacks (mostly of viral etiology) has shown the clinical benefit of MV130 as well as the protection in experimental models of respiratory viral infections by trained immunity mechanisms (Nieto et al., manuscript in preparation). Similarly, MV140 another sublingual whole cell heat-inactivated bacterial vaccine designed to prevent recurrent urinary tract infections (52, 53, 55), was also effective against urobacteria species not included in its composition (53). MV140 also triggers the release of TNFα, IL-6 and IL-1β by DCs, albeit using different signaling pathways from MV130, and induces Th1 and Th17 responses by mechanisms mediated by CLRs and TLRs as well (56). Thus, both MV130 and M140 vaccines induce the release of a similar set of cytokines ascribed to trained immunity, and favor heterologous Th1 and Th17 responses *in vivo* as described for vaccines stimulating trained immunity (26). Figure 2 summarizes the proposed action mechanisms of either MV130 or MV140 as putative TIbV. Other bacterial preparations used as nonspecific immunostimulants may also act as trained immunity inducers (see below).

### Conventional Vaccines With Associated Trained-Immunity Effects as Potential TIbV

A number of conventional anti-infectious vaccines, most of them containing live-attenuated pathogens, have been shown to induce, in addition to the intended specific memory, broad protection by nonspecific mechanisms (46, 58). Therefore, they can be considered within the category of TIbV provided that such mechanisms are related to trained immunity. If this were the case, harnessing innate memory as part of a vaccination strategy with these vaccines may be considered.

#### BCG

As above mentioned, most studies that examinated and elucidated the mechanisms of trained immunity have been performed with BCG as a model. Randomized-controlled trials carried out in Guinea-Bissau with low-birth-weight infants early vaccinated against tuberculosis with BCG, demonstrated clear beneficial effects reducing all-cause mortality, especially due to neonatal sepsis, respiratory infections, and fever (9). The effects of neonatal BCG vaccination on T and B lymphocytes subsets in infants in Denmark showed limited impact though (57) and did not affect parent-reported infections (59). It is not known whether the clinical setting in different populations, exposed to a high vs. low rate of pathogens, might account for these divergent outcomes. Recently, Arts et al. have demonstrated that BCG vaccination confers protection against viral infection (25). In a placebo-controlled clinical trial with BCG, all volunteers received the yellow fever vaccine 1 month after BCG, as an experimental mild viral infection. BCG-vaccinated volunteers displayed a significant reduction of viremia compared to the placebo group, which highly correlated with enhanced IL-1β production (25). In some experimental models of virus infection BCG immunization has been reported to confer non-specific protection; yet not in all, pointing out that the route and dose of BCG administration may be important (60).

BCG is currently used as local immunotherapy in bladder cancer (61). Interestingly, Buffen and cols. have demonstrated that the anticancer effects of BCG were dependent on trained immunity (62). In addition to a nonspecific cytotoxic effect for tumor cells by BCG-trained innate cells, these may enhance tumor specific T cell responses as a massive accumulation of tumor specific T lymphocytes are recovered in urine after successful BCG therapy (63). As endogenous tumor antigens may act as bystander antigens under the umbrella of trained immunity, this opens the possibility of TIbV as immunostimulants outside of anti-infectious purposes, e.g., tumor immunotherapy. In fact, pioneer studies of William Coley in cancer immunotherapy were based on administering bacterial products to cancer patients (64).

#### Vaccinia Virus

Live vaccinia virus was successfully used against smallpox until its eradication in 1977. Two observational studies carried out in Africa concluded that adults being smallpox vaccinated had significantly lower mortality risk, with a stronger effect observed in women than in men (65, 66). Since both studies were carried out when smallpox was already eradicated and, therefore, in the absence of the targeted infection, the beneficial effects are necessarily non-specific. The capacity of a subset of NK cells to exhibit certain aspects of innate memory following infection with vaccinia virus was found by Gillard et al. in 2011. They demonstrated that this innate memory provides host protection against a subsequent systemic infection with a lethal dose of vaccinia virus, in some cases resulting in the complete clearance of detectable virus (67).

#### Influenza Vaccine

Trivalent live attenuated influenza vaccine has been shown to confer indirect protection from respiratory illness among children (68).

Respiratory syncytial virus (RSV) and influenza virus share common features, including innate immunity activation via PRRs, such as TLR3, TLR7, and retinoic acid-inducible gene I (RIG-I) (69, 70). The cold-adapted, live attenuated influenza vaccine (CAIV) has been shown to provide non-specific cross-protection against RSV in a murine model of infection (71). The results demonstrated that this vaccination induces local immune responses that provide a broad range of antiviral immunity, including protection against RSV, and that TLR3- and TLR7- mediated innate immunity plays an important role in protection against RSV (71).

### Immunostimulants and Adjuvants as Putative TIbV

**Figure 3:**
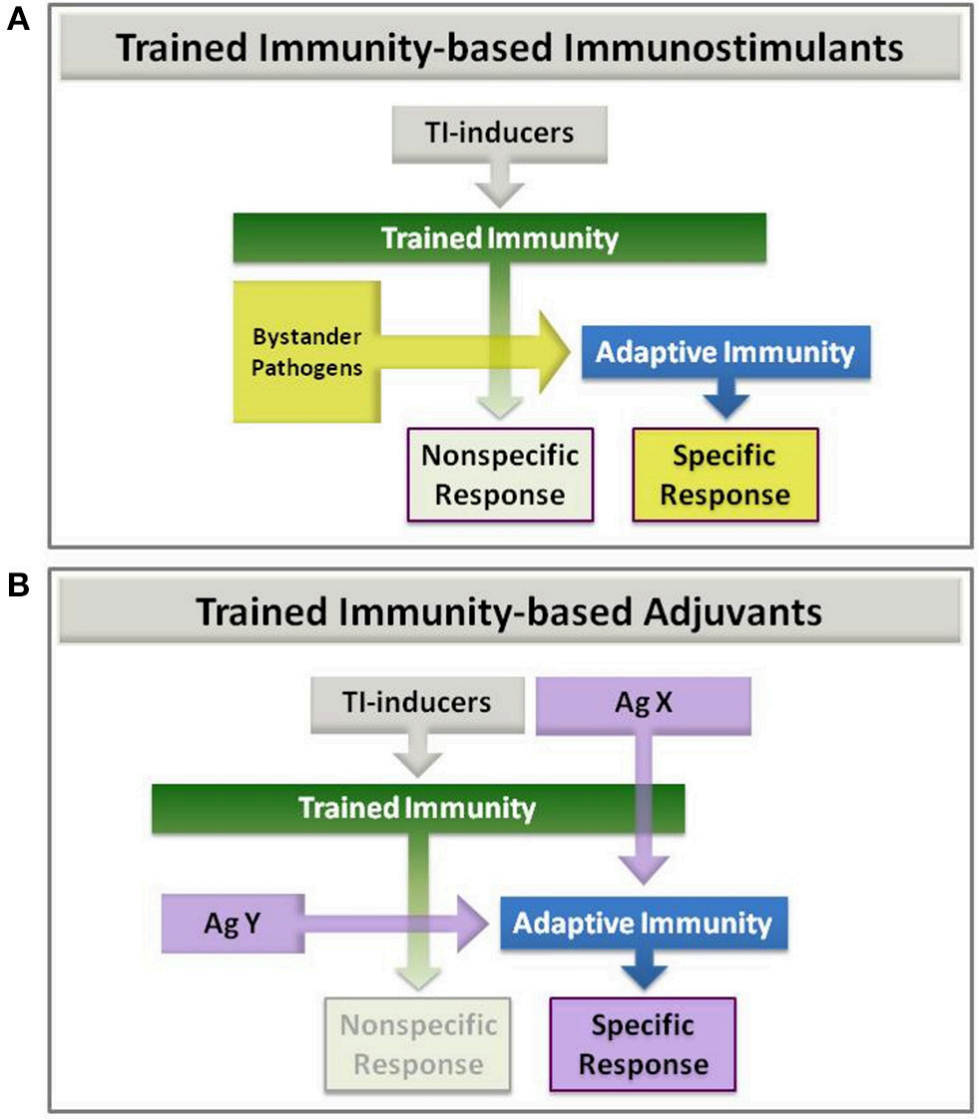
Trained Immunity-based Immunostimulants **(A)** and Adjuvants **(B)**. (A) Immunostimulants: trained immunity (TI) inducers that enhance both nonspecific and adaptive immune responses to eventual bystander pathogens. (B) Adjuvants: trained immunity (TI) inducers that are combined with an antigen. This latter can be co-delivered with the TI-inducers in the same vaccine (AgX), or later on (AgY) in a two-step process, once trained immunity has been achieved.

In addition to the examples described above, it is likely that other bacteria, fungi and viral preparations used as immunostimulants for different conditions might promote trained immunity if containing suitable inducers. In this regard, *Candida*-derived β-glucan is a paradigmatic example as it is a well-known inducer of trained immunity via dectin-1 (14). At this point, it should be noted that trained immunity-based immunostimulants might be considered within the TIbV concept because, under the umbrella of trained immunity, they may enhance innate and adaptive responses to bystander pathogens and their corresponding antigens (Figure 3A).

In 1986, Bistoni et al. demonstrated that systemic infection of mice with an avirulent *C. albicans* strain conferred protection not only against subsequent intravenous challenge with a pathogenic *C. albicans* strain but to *S. aureus* as well (72). More recently, it has been shown that protection from secondary lethal infection can be achieved with β-glucan and is dependent on epigenetic reprogramming linked to trained immunity (14). Although the immunostimulating effect of β-glucans is known for decades (73), the molecular mechanisms involved has started to be understood in the last few years (19, 21). The potential of oral β-glucan as “immune trainer” has been assayed in a pilot study in healthy volunteers (74). Innate immune responses were subsequently evaluated in peripheral blood mononuclear cells re-stimulated *in vitro* with *C. albicans*. However, the results showed a lack of cytokine production or microbicidal activity, which could be due to several reasons, including the dose and route of administration (74).

The immunostimulant OM-85 is a mixture of bacterial lysates for oral administration able to increase protection in a murine model of respiratory viral infection, reducing viral load in the lung following experimental infection (75). It also reduced rhinovirus infection of lung epithelial cells (76) and had a protective role in models of viral/bacterial respiratory infections, reducing disease symptoms and improving survival (77). OM-85 has demonstrated clinical efficacy reducing the incidence, prevalence and/or duration of infections in children and adults (78–80). It is not known whether the mechanisms behind crossprotection of this and similar immunostimulants are dependent on trained immunity, but it is likely in analogy with the bacterial vaccines described above.

Another aspect of trained immunity-based immunostimulants is that they might be considered adjuvants when combined with other antigens to which an enhanced immune response is expected (Figure 3B; AgX). Thus, TIbV containing exogenous or chimeric antigens can be furnished. This possibility has been recently described for BCG being used as adjuvant for recombinant hepatitis B surface antigen vaccination (rHBsAg) (81). Even if the antigen may be administered in a second step once the trained immunity is induced (Figure 3B; AgY). In this composition the TIbV is split in two separated elements, the trained immunity-inducer and the antigen itself. In this sense, the influence of BCG on antibody and cytokine responses to human neonatal vaccination has been described already (82). Either combination can be used for the development of novel vaccines with very specific but low antigenic molecules such as those synthetic peptides designed by reverse vaccinology.

## Clinical Applications of TIbV

The development of TIbV may represent an advantage over conventional vaccines in certain settings. Under the trained immunity umbrella, broader and stronger immune responses may be expected, without the limitations of antigen specificity. Some applications include the following:

1. When conventional vaccines are not available for pathogens that produce recurrent infections, such as those responsible for many of the respiratory and urinary infections, the most common infections in humans (83, 84). At this point, it is interesting to remark that during the 1918 influenza pandemic outbreak (Spanish flu), bacterial vaccines were used for preventive purposes with quite surprising success given the viral nature of the infection. Although this observation may be explained by a mere prevention of secondary infections of *S. pneumoniae*, which was included in many of those vaccines (85), a non-mutually exclusive alternative might be that they were acting by inducing trained immunity, i.e., as TIbV, truly protecting from flu infection.
2. To prevent illness in which bacteria and virus co-infections have a role, such as asthma exacerbations (86).
3. When directed to pathogens with high mutation rates, such as influenza virus (87, 88). The broad spectrum of TIbV may circumvent the selective pressure of highly specific vaccines. This may also be applied to avoid the emergence of new bacteria strains upon conventional vaccination as occurs with vaccines directed to specific pneumococcal serotypes (89).
4. When used for preventive purposes for individuals susceptible to infections for which there are no vaccines, i.e., children and elderly population which are susceptible to mucosal infections (90, 91). Also in certain immunodeficiency states in which innate immunity is often preserved. In this sense, TIbV might be an achievable alternative to the use of broad range of antibiotics for preventive purposes in recurrent infections (92, 93).
5. Restoring immune responsiveness in clinical conditions associated with immune paralysis, such as severe sepsis and/or malignant processes (94)

In addition, the TIbV concept can be applied to the design of vaccines directed to the antigen of interest whether it is combined or not with the trained immunity stimuli. As mentioned above this concept is also broad and TIbV may be considered immunostimulants for endogenous (bystander) pathogens, or as a type of adjuvant for any antigen. While all these applications are of primary clinical interest, caution is needed about the potential deleterious function of trained immunity in patients suffering diseases characterized by excessive inflammation. This potential deleterious effect of trained immunity could apply to atherosclerosis (95), cardiovascular events (96), gout (97), and a variety of autoimmune diseases and autoinflammatory disorders such as rheumatoid arthritis, systemic lupus erythematosus or hyper-IgD syndrome (98), where monocytes/macrophages show a detrimental trained immunity-like phenotype. Similarly, trained microglia has been linked to neurological disorders and stroke (99). Current knowledge, however, does not support such a deleterious role for TIbV: (a) Due to their nature, trained immunity is likely to have a transitory rather than a permanent effect, giving the system the required plasticity to avoid long-term potentially deleterious effects; (b) at least in two models (MV130 and MV140), TIbV have been shown to induce the production of the regulatory cytokine IL-10 by DCs and T cells, both *in vitro* and *in vivo* (47, 56); (c) MV130 has been shown to reduce recurrent infections in patients with rheumatoid arthritis without adverse effects (Candelas et al., manuscript in preparation), an observation also noted with other bacterial-derived immunostimulants (100, 101).

Many currently licensed vaccines consist of whole or inactivated pathogens; however, there has been a recent shift toward using simpler molecules such as highly purified antigens, recombinant, or synthetic peptides or DNA vaccines. Computational analysis of genetic sequences is now used for the prediction of just few T-cell epitopes fitting with most HLA molecules (102) as well as for antigen searching by the so-called reverse vaccinology (103). While these new generation of vaccines are focused to get a quite specific driven response, they are poorly immunogenic in absence of proper adjuvants. Thus, to confer protective immunity a strategy might be the combination of the adjuvant potential of trained immunity with the selected antigen epitopes. An important aspect to take into account is that it is not yet known whether or not all trained immunity stimuli produce the same functional behavior on innate immune cells with regard of driving T cells responses. As different PRR ligands may trigger different cell activation pathways with additive, synergies and opposite effects on key cell functions (44), it is likely that there are more than a single trained immunity functional program. Thus, the same antigen molecule can be eventually combined with different trained immunity stimuli for tailoring the better desired T cell response, like currently is being done with other adjuvants. In this regard, TLR8 agonists that mimic the immunomodulating effects of BCG, and enhance innate and adaptive immune responses have been described recently (104).

## Future Directions

Much knowledge is required in this field to successfully develop the potential of TIbV. Although their main advantage is that they act broadly on different pathogens and their potential as novel immunotherapy approach in both infectious, and even non-infectious immune related diseases such as cancer immunotherapy is obvious, their extent and limitations may depend on their composition. The pattern of response induced in the host innate immune cells by the specific training stimulus may also dictate the duration and interaction with ongoing specific responses. Despite the beneficial effects of TIbV inducing trained immunity as a host defense mechanism, any possible harmful effect in the induction and/or maintenance of autoimmune disorders cannot be ruled out, irrespective that current evidence does not support such a deleterious role. More studies will have to be carried out to fully understand the advantages and limitations of TIbV. Moreover, the indication of TIbV might consider several factors such as drug intake (105) or diet (106) that may affect the induction of trained immunity or its activation status, respectively. Finally, trained immunity-based stimulants and/or adjuvants widen the spectrum of the *in-silico* models for predicting immune responses. Thus, searching for new candidate combinations as trained immunity-based adjuvants for improved immunization purposes as TIbV is outlined as a novel and promising vaccine design.

## Author Contributions

SS-R, LC, and JS conducted literature searches, selected the studies, and wrote the manuscript. MN, DS, and ÓP critically contributed to the editing of the manuscript, final version and approval.

### Conflict of Interest Statement

JS is the CEO of Inmunotek SL, a pharmaceutical company that manufactures bacterial vaccines. LC is an employee of Inmunotek. SS-R, DS, and ÓP have received research grants from Inmunotek. The handling Editor declared a shared affiliation, though no other collaboration, with two of the authors ÓP and SS-R. The remaining author declares that the research was conducted in the absence of any commercial or financial relationships that could be construed as a potential conflict of interest.
